# An imminent return to drought in the western Sahel?

**DOI:** 10.1126/sciadv.adu5415

**Published:** 2025-08-20

**Authors:** Dahirou Wane, Alessandra Giannini, Alexey Kaplan, Amadou T. Gaye

**Affiliations:** ^1^ International Research Laboratory Environnement, Santé, Sociétés (IRL3189) Centre National de la Recherche Scientifique (CNRS), France and Université Cheikh Anta Diop, Dakar, Senegal.; ^2^ Laboratoire de Physique de l’Atmosphère et de l’Océan-Siméon Fongang Ecole Supérieure Polytechnique de l’Université Cheikh Anta Diop, Dakar, Senegal.; ^3^ Laboratoire de Météorologie Dynamique/IPSL Ecole Normale Supérieure, PSL Research University, Sorbonne Université Ecole Polytechnique, IP Paris, CNRS, Paris, France.; ^4^ Center for Climate Systems Research, Columbia Climate School, Columbia University, New York, NY, USA.; ^5^ Lamont Doherty Earth Observatory, Columbia University, Palisades, NY, USA.

## Abstract

In the 1970s and 1980s, the semi-arid Sahel, the southern edge of the Sahara Desert, experienced spatially uniform drought, from the Atlantic Ocean to the Red Sea. The recovery that ensued is projected to continue in the center and east, leaving the west out. We show that these two patterns—uniform variation and east-west contrast—are present in instrumental observations and in simulations with constant or time-varying external forcing. Uniform variation is amplified by 20th century external forcing, while a global warming–induced strengthening of the monsoon seeds the east-west contrast. This contrast is deepened by a mid-21st century transition to a North Atlantic cooling relative to the global tropical oceans, which affects the western Sahel most strongly because it is immediately adjacent to the North Atlantic, leading to a divergence in outcomes—between a progressively wetter central and eastern Sahel and an abruptly drier western Sahel—that is unparalleled in instrumental observations.

## Teaser

A warming-induced strengthening of the West African monsoon dries the western Sahel.

## INTRODUCTION

The climate of the Sahel, the semi-arid southern edge of the Sahara Desert, has been the focus of attention since the sudden onset of drought in the late 1960s. Research to understand drought persistence initially considered the hypothesis of a local biogeophysical feedback driven by rapid population growth (*1*) that would result in desertification. An alternative explanation linking drought to the large-scale ocean-atmosphere interaction in the Atlantic emerged shortly thereafter (*2*). It hinged on the response of the intertropical convergence zone (ITCZ) to an interhemispheric gradient in sea surface temperature (SST). Early modeling studies lent some credence to both hypotheses [e.g., (*3*, *4*)]. Conclusive evidence in favor of the dominance of oceanic influence emerged in the 2000s (*5*–*8*), when atmospheric models reproduced the 20th century evolution of Sahel rainfall based solely on the inclusion of global observed SSTs as the boundary forcing. Independent lines of evidence support this conclusion. As soon as a sufficiently long time series of satellite observations became available, it highlighted an upward trend in vegetation cover since the driest early 1980s, known as the regreening of the Sahel, which defied all notions of irreversible desertification (*9*, *10*). Most recently, environmental history studies unmasked colonial misconceptions about the role of people in shaping semi-arid landscapes (*11*).

In time, the possibility that global, rather than local, human influence may be at play gained ground. Aerosol-induced cooling of the North Atlantic (NA) emerged as an alternative explanation of 20th century Atlantic Multidecadal Variability (*12*–*14*), affecting Sahel rainfall through the latitudinal migration of the ITCZ (*15*–*19*). The role of sulfate aerosols from coal burning is further supported by modeling studies that associate large volcanic eruptions with the drying of the Sahel (*20*–*22*). The multimodel ensemble (MMM) of Phase 5 of the Coupled Model Intercomparison Project (CMIP5) simulates not only a steady post–World War II decline in Sahel rainfall but also a very dry 1982, one of the driest years in observations, in conjunction with the eruption of El Chichón (*23*, *24*).

The current consensus, therefore, is that changes in the surface temperatures of the global oceans are the dominant cause of historical Sahel drought and that these oceanic changes are partly attributable to anthropogenic emissions [partly, they are the expression of internal variability, most notably during the mid-20th century epoch of anomalously warm NA temperatures and wet Sahel that is prominent in observations (*25*–*27*)]. In other words, emissions affect Sahel rainfall indirectly, through oceanic change, which happens slowly because of the oceans’ thermal inertia, in comparison to a fast, direct change occurring through atmospheric processes (*28*–*34*). Giannini *et al.* (*28*) proposed the North Atlantic Relative Index (NARI), which is the difference between SST anomalies averaged over the subtropical NA (10° to 40°N, 75° to 15°W) and over the global tropical (GT; 20°S to 20°N) oceans, to explain 20th century variability in Sahel rainfall. Similarly to El Niño–related warming, the increase in GT stabilizes the atmosphere globally, because the warming of SSTs is communicated vertically through deep convection and is spread zonally by upper-atmosphere wave dynamics, given the weak temperature gradient approximation (*35*, *36*). Whether rains in the Sahel are abundant or deficient, then, hinges on NA, which can counter atmospheric stabilization through an increased moisture supply. Following this reasoning, Giannini and Kaplan (*24*), hereafter referred to as GK2019, attributed the late-20th century persistence of drought to the unique combination of aerosols and greenhouse gases (GHGs): Whereas GHG-induced GT warming raised the threshold for convection, aerosol-induced NA cooling reduced moisture supply.

However, SST-based arguments never worked to explain projections of future change (*24*, *37*). When analyzed separately, 20th century observations and historical simulations, on the one hand, and 20th century projections, on the other, manifest apparently irreconcilable behaviors (*38*–*40*). A spatially uniform, Sahel-wide pattern of drying and wetting dominates the past and is primarily accounted for by NARI. In contrast, future projections are characterized by opposite rainfall trends, negative in the western and positive in the central-eastern Sahel (*38*, *40*). As will be shown in detail here, these trends essentially follow global warming.

Here, we propose to answer the following questions: Can we make sense of past and future coherently? What is the role of external forcing? To what extent are the changes we see direct (atmospheric) responses, as opposed to indirect (ocean-mediated) responses to external anthropogenic forcing? In responding to these questions, we will also clarify the dynamic causes of the projected imminent transition to a more arid climate over the western Sahel.

## RESULTS

We analyze the following types of simulations from the CMIP6 archive (see Materials and Methods for details on the simulations used): historical simulations, where external forcings vary according to the best available historical estimates (Hist, 1900–2014), the high-emission scenario projection (SSP5-8.5, 2015–2100), and the so-called preindustrial control (piC).

### From uniform variation to the return of drought in the western Sahel

**Fig. 1.**
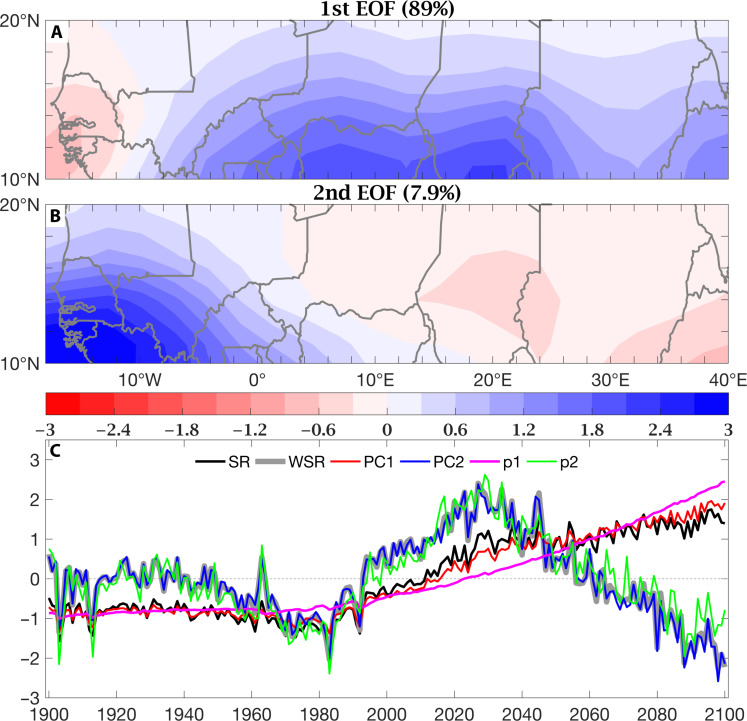
PCA of Sahel rainfall over the 1900–2100 dataset combining the MMMs of historical (1900–2014) and SSP5-8.5 (2015–2100) simulations. The two leading spatial patterns, EOF1 and EOF2, are shown in (**A**) and (**B**), respectively. Their corresponding time series, PC1 (red line) and PC2 (blue line), are shown in (**C**). (C) also includes rainfall indices for the entire Sahel (SR, black line) and its western part only (WSR, thick gray line) as well as the SST-based predictors *p*_1_ (magenta line) and *p*_2_ (green line). All time series shown in (C) are standardized for ease of comparison.

During the 20th century, Sahel rainfall was characterized by marked, largely spatially uniform, multidecadal variation (*41*), while projections show the development of a prominent east-west contrast (*38*). Looking for a coherent explanation of 20th century Sahel rainfall variations and their projected 21st century change, we start by performing empirical orthogonal function (EOF) analysis, also known as principal components analysis (PCA) [(*42*), chapter 13], on a 1900–2100 Sahel (10° to 20°N, 20°W to 40°E) precipitation dataset that combines the MMM time series of the CMIP6 Hist (1900–2014) and SSP5-8.5 (2015–2100) ensembles averaged over the July to September core of the rainy season (see Materials and Methods for details on the application of PCA). We interpret the two leading spatial patterns (EOFs) and corresponding time series [principal components (PCs)] (Fig. 1). Over the 1900–2100 period, Sahel precipitation variability is dominated by the contrast between the western (20° to 5°W, spanning Senegal and western Mali) and the central-eastern Sahel (5°W to 40°E, all the way to Ethiopia). EOF1 (Fig. 1A), with strongest loadings in the central-eastern Sahel and weaker loadings of opposite sign in the western Sahel, explains 89% of the total variance. This EOF pattern is very similar, with a pattern correlation coefficient of 0.998, to the difference between rainfall climatologies in Hist and in SSP5-8.5, since the magnitude of this jump in the mean dominates interannual to multidecadal variations. The variability captured by EOF1 is complemented by EOF2 (Fig. 1B), which shows strongest loadings over the western Sahel and explains about 8% of the total variance.

In Fig. 1C, we compare the time series of standardized July-to-September Sahel-wide (SR, black) and western Sahel (WSR, gray) rainfall with the standardized PCs associated with EOFs 1 and 2, namely, PC1 (red) and PC2 (blue). Consistent with the spatial patterns, Fig. 1C reveals that PC1 essentially tracks SR (their correlation coefficient, ρ = 0.984), while PC2 is virtually identical to WSR (ρ = 0.996). To physically interpret variations in these Sahel rainfall time series, we compare them to the PCs of the NA and GT SST time series (see Materials and Methods for details on the treatment of NA and GT). As in GK2019, the rotation of NA and GT produces a weighted sum, *p*_1_ (magenta), and a weighted difference, *p*_2_ (green), time series. The “sum,” *p*_1_, captures global warming, while the “difference,” *p*_2_, represents the oceanic influence captured in NARI. Over the whole 1900–2100 period, rainfall over the entire Sahel (PC1 and SR) is dominated by the strong increasing trend associated with *p*_1_, with progressively wetter conditions during the 21st century, but is also modulated by *p*_2_. Western Sahel rainfall (PC2 and WSR) is essentially explained by *p*_2_. Sahel-wide drought conditions between 1950 and 1990, when WSR and PC2 fall below zero, and SR and PC1 fall below *p*_1_, are associated with a strong negative phase of *p*_2_, that is, a period when the NA was persistently cooler than the GT oceans. The combination of EOFs 1 and 2 when their accompanying PCs are negative produces this uniform drying, which was most coherent during the historical drought period of the 1970s and 1980s. Similarly, the postdrought recovery is characterized by uniform Sahel wetting, as *p*_2_ (NARI) becomes positive and *p*_1_ (global warming) emerges. In time, the acceleration of global warming forces the emergence of the east-west contrast, captured in EOF/PC 1, leading to the wetting of the central-eastern Sahel and to the relative drying of the western Sahel. The drying of the western Sahel is exacerbated by the simultaneous, apparently imminent weakening of PC2 and *p*_2_, whose upward trend reverses in direction around 2030 and ultimately in sign around 2060. Overall, the same signs in *p*_1_ and *p*_2_ produce a spatially uniform response, which combines PC1 and PC2. Opposite signs in *p*_1_ and *p*_2_, specifically a *p*_1_ that trends positive with warming, and a *p*_2_ that reverses in sign, produce a markedly divergent response between the central-eastern and western Sahel, a behavior not seen in the instrumental record, which forebodes the return of drought, but only in the western Sahel.

### External influences on Sahel rainfall

**Fig. 2.**
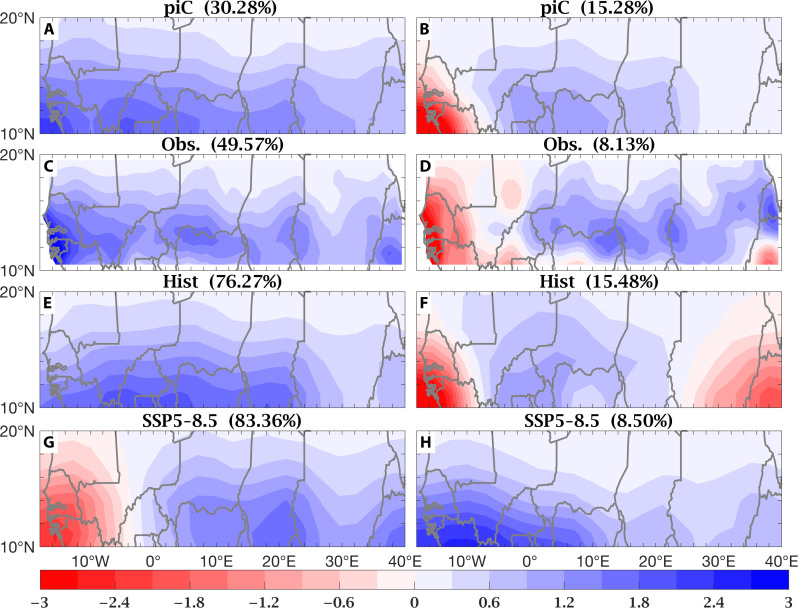
EOFs of Sahel rainfall, unitless (units of SD). The leading spatial patterns of PCA of Sahel rainfall—EOF 1 on the left and EOF2 on the right—computed separately on 200 years of the piC MMM (piC) (**A** and **B**), on observations (Obs.) over 1901–2014 (CRU) (**C** and **D**), and on the MMM of historical simulations, over 1900–2014 (Hist) (**E** and **F**) and of SSP5-8.5 projections, over 2015–2100 (SSP5-8.5) (**G** and **H**). The spatially uniform pattern dominates piC, obs, and Hist, while the east-west contrast dominates SSP5-8.5. Percent of total variance explained by each pattern is included in each panel’s title.

To understand the role of external forcing, which has transitioned from being dominated by aerosols in the second half of the 20th century to being dominated by GHGs in the 21st century, we repeat EOF analysis separately for the Hist (1900–2014) and SSP5-8.5 (2015–2100) datasets and gauge the differences in variance explained by the two leading EOFs in comparison to those in simulations with constant forcing (piC) and in observations [Climatic Research Unit (CRU), 1901–2014]. Figure 2 shows the two leading EOFs of Sahel rainfall variability computed separately for 200 years of piC (Fig. 2, A and B), for observations (Fig. 2, C and D), for Hist (Fig. 2, E and F), and for SSP5-8.5 (Fig. 2, G and H). In observations and in piC and Hist simulations, Sahel rainfall variability is dominated by the spatially uniform pattern, which explains 50, 30, and 76% of the total variance, respectively. However, a weak east-west contrast pattern is also present, explaining about 8% of the total variance in observations and 15% in piC and Hist. In SSP5-8.5, the roles of the patterns reverse: The east-west contrast becomes dominant, explaining 83% of the total variance, while the uniform pattern, which was leading in piC, Hist, and observations, is reduced to explaining only about 8.5% of the total variance (Fig. 2, G and H).

The differences in total variance explained by the spatially uniform pattern—30, 50, 76, and 8.5%, respectively, in piC, observations, Hist, and SSP5-8.5—reflect the role of external forcing. In piC, where Sahel rainfall is purely the expression of internal variability, the two leading EOFs share the total variance explained more equitably, 30 and 15%, respectively, with the uniform pattern dominating. When internal variability and external forcing combine in the single observed realization, the portion of variance explained by the uniform pattern increases to 50%, suggesting that the effects of external forcing of the historical period project onto it. When the weight of external forcing is further enhanced, by taking the MMM of Hist simulations, the portion of variance explained by this same uniform pattern increases further, to 76%.

External forcing also imprints strongly on the explanatory factors of Sahel rainfall, the GT and NA SST indices. In Table 1, we report parameters measuring their relationship, starting from correlation, as well as parameters of their linear regression of Sahel rainfall. GT and NA are only weakly correlated in piC (ρ = 0.26), but increasingly so as one moves from observations (ρ = 0.71), to historical simulations (ρ = 0.97) and to projections (ρ = 0.99). Since the portion of the signal’s variance explained by linear regression is equal to the square of its correlation skill, the bivariate regression of Sahel rainfall on *p*_1_ and *p*_2_ (or equivalently on NA and GT) only explains on the order of 10% of the total variance in Sahel precipitation, on average, in piC [ $\rho \left(y,\hat{y}\right)∼0.35$ ], compared to 35% in observations [ $\rho \left(y,\hat{y}\right)∼0.60$ ], and to 80% in historical simulations and in projections [ $\rho \left(y,\hat{y}\right)∼0.90$ ]. The latter two are substantially larger percentages than the CMIP5 values obtained in GK2019, where *p*_1_ and *p*_2_ explained 33 and 64% of the total variance, respectively, in the historical simulations and in the RCP8.5 scenario. The greater coherence between the *p*_1_ and *p*_2_ predictors and the Sahel rainfall predictand is likely due to the many large ensembles run in CMIP6, which more effectively extract the signal from noise. As in CMIP5, while warming dominates the 21st century, especially in the central-eastern Sahel, NARI holds more explanatory power in the 20th century, in observations as well as in historical simulations, and especially so in the western Sahel. As discussed above, we expect *p*_1_ to capture spatially coherent warming and cooling of the 20th century. In contrast, *p*_2_ (NARI) captures the enhanced post–World War II cooling of the NA attributed to aerosols, as GHGs began to warm the tropical oceans. The switch to the dominance of the east-west contrast in SSP5-8.5 is another sign that the external forcing has changed to one that strongly favors warming.

**Table 1.** Parameters characterizing the relationship of NA and GT and that of their PCs, namely, *p*_1_ and *p*_2_, to central-eastern and western Sahel rainfall. Columns from left to right show the dataset analyzed (observations or CMIP6 simulations), the parameters describing the relationship of GT and NA, on the left, including their correlation coefficient (ρ), the ratio of their SDs [κ = σ(GT)/σ(NA)], the angle (ϕ, in arc degree) of the PCA rotation of GT and NA, the percent of total variance explained by the trailing mode with respect to the sum of leading and trailing modes, λ_1_ and λ_2_, where the leading and trailing modes correspond to time series *p*_1_ and *p*_2_, respectively, and parameters describing the regression model $\hat{y}=ap_{1}+bp_{2}$ , on the right, where *y* is rainfall in the central-eastern (CES) or western (WS) Sahel, and the two rightmost columns represent the correlation of Sahel rainfall as directly output from simulations and as modeled in the regression.

	ρ	κ	ϕ	$\frac{\lambda_{2}}{\lambda_{1}+\lambda_{2}}$	*a*		*b*		ρ(*y*,$\hat{y}$)	
					CES	WS	CES	WS	CES	WS
piControl (200 years)	0.26	1.02	42.93	36.78	−0.23	−0.07	0.22	0.38	0.32	0.38
CRU/Kaplan (1901–2014)	0.71	0.79	54.28	13.40	−0.07	−0.10	0.56	0.62	0.57	0.63
Historical (1900–2014)	0.97	0.94	46.89	1.33	0.84	0.30	0.39	0.85	0.92	0.90
SSP5-8.5 (2015–2100)	0.99	1.14	41.33	0.03	0.83	−0.95	0.06	0.24	0.83	0.96
CMIP6 (1900–2100)	0.99	1.04	43.80	0.10	0.97	−0.50	0.22	0.80	0.99	0.95

### Monsoon strengthening and the divergence between western and central-eastern Sahel futures

**Fig. 3.**
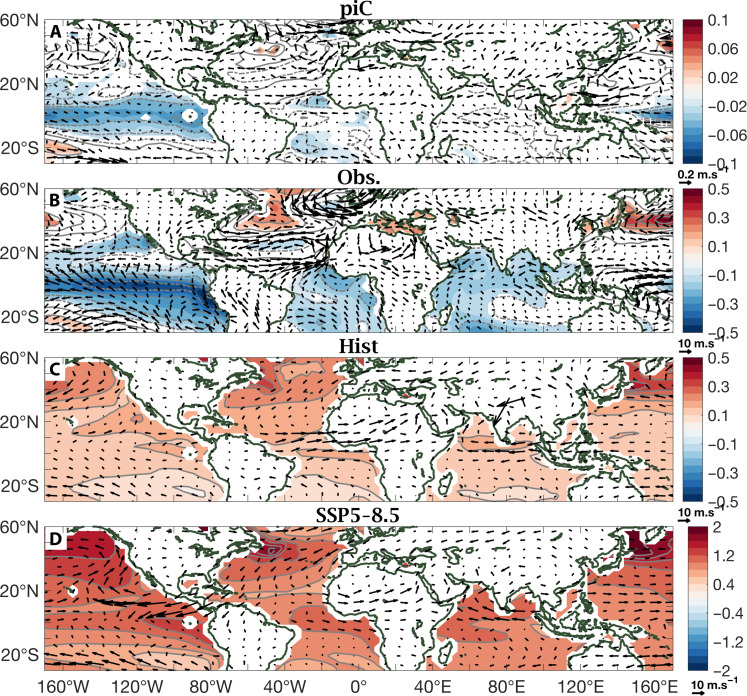
Regressions on PC1 of Sahel rainfall. Regressions of SST (gray contours and color shading) and low-level winds (vectors) on the PC1 time series computed on the MMM of piC simulations (**A**), on observations (Obs.) (**B**), and on the MMMs of historical simulations (Hist) (**C**) and of future high-emission scenario projections (SSP5-8.5) (**D**). SST regression values are represented in the gray contours, solid if positive, and dashed if negative, and in color shading, if values are significant at 95% level. They are plotted every 0.02°C for piC, 0.1°C for Obs. and Hist, and 0.2°C for SSP5-8.5. Note that the color scale ranges vary.

**Fig. 4.**
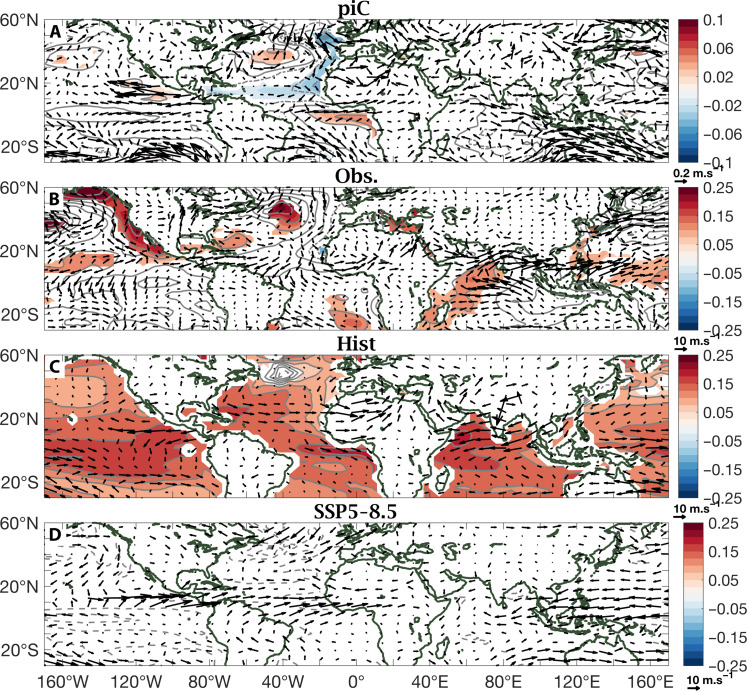
Regressions on PC2 of Sahel rainfall. (**A** to **D**) Same as Fig. 3 but for PC2. Note that the absence of color shading is indicative of a lack of statistical significance of the SST regression values.

The dynamics linking Sahel rainfall variability to external forcing can be elucidated by regressing SST and low-level winds onto the PCs of Sahel rainfall (PC1 in Fig. 3 and PC2 in Fig. 4) in each configuration: piC, observations, Hist, and SSP5-8.5. The regression patterns in piC and in observations (see Materials and Methods for details) are broadly similar (see Figs. 3, A and B, and 4, A and B). In the absence of time-varying external forcing to synchronize anomalies across models, anomalies in piC, the expression of the random assemblage of independent internal variability in each model realization, are an order of magnitude weaker. For piC and observations, positive anomalies in PC1, the amplitudes of the uniform pattern of Sahel precipitation variability, are associated with cooler global tropics, including in the El Niño–Southern Oscillation region of the tropical Pacific, and a warmer subtropical NA, that is, with the constituent elements of NARI (Fig. 3, A and B). The consequent strengthening of the monsoon is visible in southwesterly wind anomalies across the entire Sahel. In Hist (Fig. 3C), the signature of warming is dominated by its more recent phase, that of excessive NA warming, which results in a positive NARI. This warming is understood to be the consequence of the marked reduction in aerosol emissions over North America and Europe that followed legislation to counter their effects on the environment and public health (*43*). In this regard, the pattern differs substantially not only from piC and observations but also from CMIP5 [GK2019; (*27*)]. This is possibly due to differences in the sensitivity of the different CMIP generations to external forcing (*44*, *45*).

The difference between Hist and SSP5-8.5 regression patterns associated with the respective PC1’s, underlying a uniform Sahel pattern in Hist (Fig. 3C) and east-west contrast in SSP5-8.5 (Fig. 3D), is all in a subtle difference in the direction of the winds, and the moisture advection that they embody, at the western edge of the Sahel. Southwesterly winds in Hist wet the western Sahel: They advect air that is relatively high in moist static energy (MSE) content because it is climatologically warmer and/or more humid. Northwesterly winds in SSP5-8.5 dry it, as they advect air that is relatively low in MSE content, being climatologically cooler and/or less humid. Northwesterly winds at the west coast of West Africa are also visible in Fig. 4 (A to C); that is, they are associated with PC2, the east-west contrast in piC, observations, and Hist. Here, too, they dry the western Sahel. Last, the last regression map, that of PC2 in SSP5-8.5 (Fig. 4D), represents a more localized mechanism that can further separate the western Sahel from the rest. Southwesterly anomalies at the West African coast, which would be associated with a wet western Sahel, are reminiscent of the coupled ocean-atmosphere dynamics described in (*46*) on subseasonal time scales. Here, since PC2 is projected to reverse to negative in mid-century, they represent northeasterly anomalies in the same region, which would push away moisture from the continent, further drying its western edge.

## DISCUSSION

The east-west contrast, with strong positive loadings in the central-eastern Sahel and negative loadings in the western Sahel, dominates the 21st century and the mean change between the 20th and 21st centuries. It stands in stark contrast to past behavior, which was characterized by spatially uniform variation: drying during the post–World War II multidecadal drought episode that culminated in the 1970s and 1980s and wetting during the partial recovery since. Since its associated time series, PC1 depicts a positive trend that strengthens in time, and we interpret this pattern to express the direct/atmospheric response of the West African monsoon to warming: As the planet warms, the circulation around the Saharan heat low strengthens. Relatively high-moisture air is advected farther poleward in the central-eastern Sahel, while relatively low-moisture air comes onshore in the western Sahel. Western Sahel drying is amplified when warming, as expressed in *p*_1_, is accompanied by a switch in sign in *p*_2_ (Fig.1C). The imminent weakening of *p*_2_ and its full reversal to negative values projected by the middle of the 21st century are a cause for concern, since it could return the western Sahel to drought. However, while an east-west contrast in the recovery of the rains has been noted in observations (*47*), strong trends have not emerged either way. The character of the rainy season in recent decades has been described as hybrid: overall wetter in accumulation, but more erratic, that is, with more false starts and early cessations, and a greater frequency of daily extremes (*48*). The same holds true for projections (*49*). In addition, the current epoch appears to be characterized by greater interannual variability overall, as the constituent elements of NARI, NA, and GT compete in warming (*28*). Furthermore, the magnitude of the direct response to warming that emerges as circum-NA aerosol concentrations abate is stronger in CMIP6 than in CMIP5 and controversially so.

In sum, we are confident that the present analysis clearly identifies the constituent elements of Sahelian climate change—an east-west contrast fueled by the fast/direct/atmospheric response to warming and a stronger western Sahelian dependence on the slow/indirect/oceanic response expressed in relative NA warming. However, we must note that the weight of the relative contributions of the two mechanisms to uncertainty in projections remains indeterminate. Future work that considers similarities and differences in these relative contributions across the multimodel ensemble could help constrain model uncertainties better.

Since the sudden onset of persistent drought in the late 1960s, our knowledge of West African monsoon dynamics has come a long way; so has its simulation in climate models, including in the climate prediction context. We offer the predictive elements described here to enable discussion of policies that anticipate and reduce societal impact. We seek to move the discussion away from equating climate change with drought in the Sahel (consistently with paleoclimate reconstructions of a Green Sahara 6000 years ago), albeit with the caveat of a possible imminent—on decadal timescale—return of drought in the western Sahel.

## MATERIALS AND METHODS

### Climate model output

We use output from a subset of model simulations contributed to the CMIP in support of the Sixth Assessment Report of the Intergovernmental Panel on Climate Change, referred to as CMIP6 (*50*). Table S1 lists the CMIP6 models we used, their ensemble sizes, and the time periods covered by each type of simulation. We restrict our analysis to 13 CMIP6 models whose individual ensembles had at least five Hist simulations. About half of the selected models had so-called large ensembles of Hist simulations containing 25 or more members (table S1). We use three types of CMIP6 simulations: piC, where external forcing (top of the atmosphere, or TOA, insolation, and CO_2_ concentration) is held fixed in time for the entire simulation; thus, all the simulated variability is internal to the system; historical (Hist), where external forcings vary according to the best available observational estimates; and the high-emission scenario projection (SSP5-8.5). While other scenario projections are now considered to be more realistic, we choose to analyze the high emission scenario, with an end of 21st century TOA radiative imbalance of 8.5 W/m^2^, to ensure that a strong climate response can be detected and interpreted. For each type of simulation, we first calculate the means of each model’s own ensemble and then average these to obtain the mean of the MMM. We focus on the MMM because we are interested in the robust component of Sahel rainfall response to external forcing, that which is shared by all models—whether the forcing is natural, that is, due to variations in TOA insolation and injection of aerosols from volcanic eruptions, or anthropogenic, that is, most notably, due to emissions of aerosols and GHGs from fossil fuel burning.

### Observations

To validate historical CMIP6 simulations and to assess the relative importance of internal variability and external forcing, we compare model output to the following observational datasets over the 1901 to 2014 period: gridded monthly precipitation from the CRU (*51*), gridded monthly SST from Kaplan (*52*), and surface winds from the Twentieth Century Reanalysis (*53*).

### Principal components analysis

Also called EOF analysis, it is performed by finding the leading eigenvectors of the covariance matrix of the data or by performing singular value decomposition directly on the data matrix (*42*). When the data represent spatiotemporal variability, each mode is associated with a single spatial pattern and a single time series of coefficients that scale the contribution of each pattern to the total field at each time. The patterns, called EOFs, form an orthonormal set, while the corresponding time series, called PCs, are orthogonal to each other and have unequal variances, precisely those explained by the corresponding modes. In climate science, the procedure is useful when the dominant modes of variability can be interpreted in terms of physical processes, e.g., see (*5*, *54*) in the case of West African precipitation and related mechanisms.

Here, we apply PCA to observed and modeled (MMMs from selected CMIP6 experiments) precipitation fields over a Sahelian domain, defined broadly to encompass all latitude points between 10° and 20°N and all longitude points between 20°W and 40°E, during the July to September core of the rainy season. Results are displayed in Figs. 1 and 2. To explain the underlying physical mechanisms, we compute patterns of linear regression coefficients of the global SST and low-level wind fields on the Sahel rainfall PC time series, PC1 and PC2. Results are displayed in Figs. 3 and 4. Both PC1 and PC2 of Sahel rainfall and the regression patterns on them of SSTs and low-level winds were calculated using MMM fields. The 5% statistical significance is assessed using a Monte Carlo test, permuting the time series 1000 times.

### Orthogonal SST-based predictors

*p*_1_ and *p*_2_ are derived as standardized PCs of a dataset from a two-dimensional space spanned by SST indices (GT and NA). Because the space dimension is so low, there is a closed-form expression for *p*_1_ and *p*_2_, which shows that a two-dimensional vector (*p*_1_ and *p*_2_) is obtained from a vector (NA and GT) by a rotation to a certain angle ϕ followed by a rescaling with coefficients $\sqrt{\lambda_{1}}$ and $\sqrt{\lambda_{2}}$ , which are the SDs of the variability modes corresponding to *p*_1_ and *p*_2_. These transformation parameters (ϕ, λ_1_, and λ_2_) are expressed through SDs of GT and NA and a correlation coefficient between them (GK2019). Since the leading mode captures most of the variance, it is not unexpected that in the case of GT and NA based on SST observations, historical simulations, and high emission scenario projections, *p*_1_ represents the common warming trend, a weighted sum of NA and GT. Conversely, the second mode, being constrained by the orthogonality requirement, is a weighted difference of NA and GT (*p*_2_ ~ NARI). The parameters that describe the relationship of NA and GT time series, namely, their correlation coefficient ρ(GT, NA) and the ratio of their SDs κ = σ(GT)/σ(NA), let us calculate the required angle of rotation (ϕ) to produce *p*_1_ and *p*_2_ and the resulting percentage of total variance explained by the trailing mode *p*_2_: $\lambda_{2}/\left(\lambda_{1}+\lambda_{2}\right)*100\%$ . Here, we update for the CMIP6 case the analysis performed by GK2019 for a CMIP5 ensemble. We report the parameters described above, for the MMM (Table 1) and for each model’s individual ensemble mean (tables S2 to S5).

The main application of the *p*_1_ and *p*_2_ time series is the prediction of Sahel rainfall. For the standardized rainfall timeseries *y*, the prediction formula is $\hat{y}=ap_{1}+bp_{2}$ , where regression coefficients *a* and *b* are found as correlation coefficients of *p*_1_ and *p*_2_ with *y*. The regression coefficients *a* and *b*, together with the correlation coefficients between *y* and its prediction $\hat{y}$ that characterizes the regression skill, are reported in Table 1 and tables S2 to S5.
