# Supplementary material for: An imminent return to drought in the western Sahel?
Source: Sci Adv. 2025 Aug 20;11(34):eadu5415. doi: 10.1126/sciadv.adu5415 (PMC12366705; doi:10.1126/sciadv.adu5415)
Supplement: Supplementary file 1 — Tables S1 to S5 [file sciadv.adu5415_sm.pdf]

Science Advances
AAAS

Supplementary Materials for

An imminent return to drought in the western Sahel?

Dahirou Wane *et al.*

Corresponding author: Dahirou Wane, dahirou.wane@ucad.edu.sn

Sci. Adv. **11**, eadu5415 (2025)
DOI: 10.1126/sciadv.adu5415

This PDF file includes:

Tables S1 to S5

table S1: **Sizes of individual model ensembles from CMIP6** pre-industrial control (piC), historical (Hist), and high-emission scenario (SSP5-8.5) experiments used in this work. The names of the six models for which historical experiments were so-called “large ensemble simulations” (with 25 or more ensemble members) are shown in bold type.

CMIP6 Models	piC [200 years]	Hist [1900–2014]	SSP5-8.5 [2015–2100]
**ACCESS-ESM1-5**	1	30	30
CESM2	1	11	5
**CNRM-CM6-1**	1	26	6
**CanESM5**	1	25	25
FGOALS-g3	0	6	4
GISS-E2-1-H	1	10	5
HadGEM3-GC31-LL	1	5	4
INM-CM5-0	1	10	1
**IPSL-CM6A-LR**	1	32	6
**MIROC-ES2L**	1	30	10
**MIROC6**	1	50	50
MPI-ESM1-2-LR	1	10	10
MRI-ESM2-0	1	5	5
Multi-model mean	12	13	13

table S2: **Parameters characterizing the relationship of NA and GT, and that of their PCs, namely p_1 and p_2, to central-eastern and western Sahel rainfall in the concatenation of Hist and SSP5-8.5.** Columns from left to right show model name, the parameters describing the relationship of GT and NA, on the left, including their correlation coefficient (ρ), the ratio of their standard deviations ($\kappa = \sigma_1/\sigma_2$), where subscripts 1 and 2 refer to GT and NA, respectively, the angle (ϕ, in arc degree) of the PCA rotation of GT and NA, the percent of total variance explained by the trailing mode with respect to the sum of leading and trailing modes, λ_1 and λ_2, where the leading and trailing modes correspond to time series p_1 and p_2, respectively, and parameters describing the regression model $\hat{y} = ap_1 + bp_2$, on the right, where y is rainfall in the central-eastern (CES) or western (WS) Sahel, and the two rightmost columns represent the correlation of Sahel rainfall as directly output from simulations and as modeled in the regression.

	ρ	κ	ϕ	$\frac{\lambda_2}{\lambda_1+\lambda_2}$	a		b		$\rho(y,\hat{y})$	
					CES	WS	CES	WS	CES	WS
ACCESS-ESM1-5	0.99	1.04	43.94	0.28	0.84	-0.50	0.44	0.76	0.95	0.91
CESM2	0.99	1.33	36.95	0.34	0.15	-0.81	0.64	0.46	0.66	0.93
CNRM-CM6-1	1.00	0.95	46.40	0.10	0.85	-0.43	0.20	0.46	0.87	0.63
CanESM5	1.00	0.97	45.95	0.07	0.99	0.64	0.07	0.50	0.99	0.81
FGOALS-g3	0.99	1.22	39.31	0.32	0.21	-0.93	0.43	0.11	0.47	0.94
GISS-E2-1-H	0.99	1.15	40.99	0.41	0.32	-0.77	0.35	0.25	0.47	0.81
HadGEM3-GC31-LL	1.00	1.04	43.88	0.27	0.89	-0.73	0.18	0.35	0.91	0.81
INM-CM5-0	0.99	0.94	46.72	0.65	0.74	0.64	0.18	0.26	0.76	0.69
IPSL-CM6A-LR	1.00	1.01	44.65	0.17	0.93	-0.58	0.22	0.56	0.96	0.80
MIROC-ES2L	1.00	1.01	44.84	0.15	0.91	-0.11	0.14	0.47	0.92	0.48
MIROC6	1.00	0.93	47.17	0.13	0.97	-0.72	0.19	0.53	0.99	0.89
MPI-ESM1-2-LR	0.99	0.98	45.62	0.29	0.96	0.81	0.13	0.38	0.97	0.90
MRI-ESM2-0	0.99	1.09	42.39	0.74	0.45	-0.61	0.57	0.59	0.73	0.85
Multi-model mean	1.00	1.04	43.80	0.10	0.97	-0.50	0.22	0.80	0.99	0.95

table S3: **Parameters characterizing the relationship of NA and GT, and that of their PCs, namely p_1 and p_2, to central-eastern and western Sahel rainfall in the pre-industrial control simulations.** See the caption in table S2 for details.

	ρ	κ	ϕ	$\frac{\lambda_2}{\lambda_1+\lambda_2}$	a		b		ρ(y,ŷ)	
					CES	WS	CES	WS	CES	WS
ACCESS-ESM1-5	0.29	1.08	37.30	35.18	-0.38	-0.17	0.23	0.41	0.45	0.45
CESM2	0.38	1.04	42.29	31.05	-0.34	-0.21	0.38	0.49	0.51	0.53
CNRM-CM6-1	0.37	0.80	60.72	29.00	0.08	0.15	0.25	0.36	0.26	0.39
CanESM5	0.36	0.94	49.93	31.84	0.09	-0.01	0.03	0.13	0.09	0.13
GISS-E2-1-H	0.33	1.52	18.87	25.00	0.24	0.09	-0.01	0.23	0.24	0.25
HadGEM3-GC31-LL	0.26	0.65	75.06	26.52	-0.04	0.35	0.21	0.29	0.21	0.46
INM-CM5-0	0.36	0.56	74.74	19.64	-0.05	0.21	0.25	0.13	0.25	0.25
IPSL-CM6A-LR	0.37	0.85	57.15	30.21	-0.11	0.09	0.38	0.42	0.40	0.43
MIROC-ES2L	0.54	1.68	22.45	16.40	-0.16	-0.29	-0.03	0.26	0.16	0.39
MIROC6	0.48	1.54	23.45	20.10	-0.29	-0.28	0.08	0.35	0.30	0.45
MPI-ESM1-2-LR	0.46	1.16	35.86	25.96	-0.57	-0.28	0.30	0.48	0.64	0.56
MRI-ESM2-0	0.38	0.71	66.01	25.58	-0.04	0.26	0.09	0.20	0.10	0.32
Multi-model mean	0.26	1.02	42.93	36.78	-0.23	-0.07	0.22	0.38	0.32	0.38

table S4: **Parameters characterizing the relationship of NA and GT, and that of their PCs, namely p_1 and p_2, to central-eastern and western Sahel rainfall in the Historical simulations.** See the caption in table S2 for details.

	ρ	κ	ϕ	$\frac{\lambda_2}{\lambda_1+\lambda_2}$	a		b		ρ(y,ŷ)	
					CES	WS	CES	WS	CES	WS
ACCESS-ESM1-5	0.93	0.84	50.49	3.46	0.69	0.21	0.47	0.75	0.84	0.78
CESM2	0.91	0.91	47.87	4.28	0.62	0.22	0.42	0.75	0.75	0.76
CNRM-CM6-1	0.96	0.84	50.12	1.74	0.84	0.73	0.04	0.19	0.84	0.93
CanESM5	0.97	1.02	44.48	1.46	0.94	0.89	0.05	0.25	0.94	0.75
FGOALS-g3	0.95	1.08	42.62	2.42	0.41	-0.67	0.18	0.34	0.45	0.26
GISS-E2-1-H	0.88	1.19	39.31	5.63	0.64	-0.09	-0.09	0.25	0.64	0.46
HadGEM3-GC31-LL	0.87	0.79	52.59	6.30	0.64	0.09	0.20	0.45	0.67	0.41
INM-CM5-0	0.90	0.99	45.23	5.26	0.16	0.16	0.31	0.38	0.35	0.69
IPSL-CM6A-LR	0.97	0.84	50.30	1.55	0.74	0.37	0.40	0.58	0.85	0.47
MIROC-ES2L	0.94	0.92	47.55	2.87	0.40	-0.01	0.36	0.47	0.54	0.78
MIROC6	0.91	0.97	45.96	4.64	0.60	-0.06	0.60	0.78	0.85	0.70
MPI-ESM1-2-LR	0.92	0.91	47.99	3.78	0.60	0.53	0.33	0.46	0.68	0.63
MRI-ESM2-0	0.83	0.79	53.05	8.09	0.34	0.18	0.45	0.61	0.56	0.90
Multi-model mean	0.97	0.94	46.89	1.33	0.84	0.30	0.39	0.85	0.92	0.90

table S5: **Parameters characterizing the relationship of NA and GT, and that of their PCs, namely p_1 and p_2, to central-eastern and western Sahel rainfall in the SSP5-8.5 projections.** See the caption in table S2 for details.

	ρ	κ	ϕ	$\frac{\lambda_2}{\lambda_1+\lambda_2}$	*a*		*b*		ρ(y,ŷ)	
					CES	WS	CES	WS	CES	WS
ACCESS-ESM1-5	1.00	1.22	39.25	0.10	0.41	-0.94	0.38	0.16	0.55	0.96
CESM2	0.99	1.51	33.42	0.29	-0.61	-0.93	0.10	0.20	0.61	0.94
CNRM-CM6-1	1.00	0.99	45.24	0.18	0.54	-0.83	0.15	0.13	0.56	0.84
CanESM5	1.00	1.02	44.36	0.04	0.98	-0.66	0.07	0.38	0.98	0.77
FGOALS-g3	0.98	1.31	37.33	0.72	-0.40	-0.85	0.29	0.28	0.50	0.89
GISS-E2-1-H	0.99	1.30	37.41	0.61	-0.62	-0.86	0.10	0.06	0.63	0.86
HadGEM3-GC31-LL	0.99	1.14	41.33	0.33	0.58	-0.75	0.07	0.33	0.58	0.82
INM-CM5-0	0.96	1.01	44.76	2.24	0.42	0.27	0.20	0.28	0.47	0.40
IPSL-CM6A-LR	0.99	1.06	43.31	0.32	0.81	-0.73	0.29	0.42	0.87	0.83
MIROC-ES2L	0.99	1.04	43.88	0.30	0.77	-0.28	0.20	0.44	0.79	0.52
MIROC6	1.00	0.98	45.71	0.07	0.95	-0.91	0.13	0.19	0.96	0.93
MPI-ESM1-2-LR	0.99	1.08	42.89	0.28	0.91	0.44	0.19	0.44	0.94	0.63
MRI-ESM2-0	0.99	1.35	36.52	0.61	-0.56	-0.87	0.19	0.24	0.59	0.90
Multi-model mean	1.00	1.14	41.33	0.03	0.83	-0.95	0.06	0.24	0.83	0.96
